# The EP-VRD2 for endovascular treatment of ophthalmic segment aneurysms: wall apposition and long-term study

**DOI:** 10.3389/fneur.2026.1750959

**Published:** 2026-03-26

**Authors:** Jinlong Yuan, Chenlei Huang, Zhenbao Li, Niansheng Lai, Jiaqiang Liu, Bingbing Zhang, Gang Zhou, Dayong Xia, Xintong Zhao

**Affiliations:** 1 The Translational Research Institute for Neurological Disorders of Wannan Medical College, Department of Neurosurgery, The First Affiliated Hospital of Wannan Medical College (Yijishan Hospital of Wannan Medical College), Wuhu, China; 2 Department of Clinical Laboratory, The First Affiliated Hospital of Wannan Medical College (Yijishan Hospital of Wannan Medical College), Wuhu, China

**Keywords:** ophthalmic segment aneurysms, endovascular treatment, EP-VRD2, incomplete stent apposition, wall apposition

## Abstract

**Objectives:**

The Enterprise Vascular Reconstruction Devices 2 (EP-VRD2) are designed to improve apposition to curved vessel walls. This study aimed to evaluate the wall apposition (WA) and long-term safety and effectiveness of EP-VRD2 for treating ophthalmic segment aneurysms (OSAs).

**Materials and methods:**

Dyna CT angiography (CTA) was used to evaluate WA, while digital subtraction angiography (DSA) and the modified Rankin score (mRS) were utilized for imaging and outcome follow-up, respectively. The minimum follow-up was 12 months.

**Results:**

A total of 104 OSAs treated with EP-VRD2 were collected. The complication rate was 3.84%, all due to thromboembolic events. Immediate angiography revealed Raymond–Roy occlusion classification (RROC) I in 73.08% of cases, II in 23.08%, and III in 3.84%. DSA follow-up of 90 OSAs over an average of 19.91 ± 7.16 months showed a complete occlusion rate of 95.56%. Clinical follow-up averaged 22.15 ± 9.26 months, with mRS ranging from 0 to 2. Crescent signs, indicating incomplete stent apposition (ISA), were observed in 11.54% of cases. Curvature radius (CR) and artery angle (AA) were significantly different between the complete apposition and ISA groups, with cut-off values identified at 44° and 3.58 mm.

**Conclusion:**

EP-VRD2 stents are safe and effective for long-term treatment of OSAs, significantly improving WA in curved vessels. However, ISA remains in sharp ICA siphon bends, particularly with an AA < 44° or CR < 3.58 mm.

## Introduction

The Ophthalmic segment aneurysms (OSAs) are located between the distal dural ring and the origin of the posterior communicating artery, accounting for approximately 5–10% of intracranial aneurysms (IAs) (1, 2). Due to the complex anatomy, limited intraoperative exposure, and the need to grind down the anterior clinoid process and retract the optic nerve, microsurgical clipping poses a significant technical challenge for neurosurgeons. However, with the development of interventional materials and technology, particularly the application of stent-assisted coil (SAC) technology and flow diverters (FDs), the effectiveness of treating OSAs has improved substantially (3, 4). Moreover, endovascular treatment is minimally invasive and associated with low complication rates, making it the preferred method for treating OSAs.

The EP-VRD (Enterprise Vascular Reconstruction Device, Codman Neurovascular, Raynham, MA, USA) was the first self-expanding laser-cut device with a closed-cell design. Although several studies have shown that the EP-VRD effectively treats intracranial wide-necked aneurysms, ischemic events caused by incomplete stent apposition (ISA) remain a challenge (5–7). For OSAs, deploying stents and achieving complete apposition (CA) to the vessel wall is more challenging due to the need to pass through the curved internal carotid artery (ICA) siphon segment, which is constrained by the skull. To address this shortcoming, the EP-VRD2 (Codman Neurovascular, Raynham, MA, USA) was developed. The device features several design improvements. First, the maximum opening diameter was increased from 4.5 to 5 mm. Second, the EP-VRD2 has a higher amplitude of the sinusoidal wave. These structural changes facilitate greater elongation on the outer radius of the curvature and compression on the inner radius, both of which enhance wall apposition (WA) performance. Some studies have also found that the WA performance of the EP-VRD2 is superior to that of the EP-VRD *in vivo* and has achieved favorable short-term results (8, 9). Such short-term research may lead to inaccurate conclusions. Therefore, we conducted this retrospective study to assess the long-term safety, efficacy, and wall apposition (WA) performance of the EP-VRD2 stent in the treatment of OSAs. To the best of our knowledge, this study represents the largest sample of its kind.

## Materials and methods

Participants were selected from the Department of Neurosurgery at the First Affiliated Hospital of Wannan Medical College. The study was approved by the hospital’s Research Ethics Committee and conducted in accordance with the Helsinki Declaration. Informed consent was obtained from all participants or their families.

### Patient selection

Patients with OSAs who underwent EP-VRD2 treatment between January 2019 and January 2024 were included. During this period, a total of 218 patients with OSAs were treated at our center. The decision to use EP-VRD2 followed a standardized institutional protocol. EP-VRD2-assisted coiling was the preferred strategy for saccular aneurysms that were anatomically suitable for stent delivery and in patients eligible for dual antiplatelet therapy. As reflected in our cohort (Table 1), this protocol primarily selected patients with small-to-medium aneurysms (mean size 3.82 mm; 96.15% < 10 mm) and unfavorable dome-to-neck ratios (96.15% < 2). Patients with fusiform/blister morphology, giant aneurysms, or contraindications to antiplatelet therapy were typically directed toward alternative treatments (e.g., FDs, other stents, or surgical clipping).

**Table 1:** Characteristics of 104 patients with OSAs treated with EP-VRD2.

Characteristics	Results
Age (mean ± SD)	59.17 ± 11.52
Female (*n*, %)	78 (75%)
Multiple aneurysms (*n*, %)	30 (28.85%)
Hypertension (*n*, %)	38 (36.59%)
Diabetes mellitus (*n*, %)	10 (9.62%)
Cerebral infarction (*n*, %)	14 (13.46%)
Subarachnoid hemorrhage (*n*, %)	16 (15.38%)
I	2
II	12
III	2
IV	0
V	0
Presentation (*n*, %)	88 (84.62%)
Headaches	20 (19.23%)
Dizziness	32 (30.77%)
Blurred vision	6 (5.77%)
Asymptomatic	20 (19.23%)
History of IAs operation	10 (9.62%)
Right site (*n*, %)	40 (38.46%)
Size (mm)	3.82 ± 2.84
<7	92 (88.46%)
7–10	8 (7.69%)
>10	4 (3.85%)
Neck width	3.62 ± 1.81
Wide (≥4 mm)	36 (34.62%)
Narrow (<4 mm)	68 (65.38%)
Dome-to-neck ratio	1.18 ± 0.30
Favorable (≥2)	4 (3.85%)
Unfavorable (<2)	100 (96.15%)

IAs, intracranial aneurysms.

From this overall cohort, 104 consecutive patients (47.7%) who met the inclusion criteria and were treated with EP-VRD2 constituted the study group. Inclusion criteria were: (1) OSAs diagnosed by CTA, MRA, or DSA; (2) EP-VRD2-assisted coil treatment through the curved ICA siphon; (3) complete clinical and imaging data; and (4) follow-up time of ≥12 months. Exclusion criteria included: (1) non-saccular aneurysms; (2) arteriovenous malformations, moyamoya disease, or intracranial tumors; (3) simple coil embolization or surgical clipping; and (4) incomplete data. A total of 104 consecutive patients were included, with aneurysms categorized by size as <7 mm, 7–10 mm, or ≥10 mm.

### Perioperative antiplatelet therapies

For unruptured OSAs, patients received aspirin (100 mg/day) and clopidogrel (75 mg/day) for at least 3 days before surgery, followed by thromboelastography. If the inhibition rate was <30% for arachidonic acid or adenosine diphosphate (ADP), aspirin was switched to cilostazol and clopidogrel to ticagrelor, respectively, with repeat testing. All patients met predefined antithrombotic standards (arachidonic acid >30% and ADP > 30%) before EP-VRD2 treatment. For ruptured OSAs, patients received loading doses of aspirin (300 mg) and clopidogrel (300 mg) 2 h before anesthesia. Postoperatively, all patients continued aspirin (100 mg/day) and clopidogrel (75 mg/day) for 6 weeks, followed by aspirin alone for 1 year.

### Procedure

The procedure was performed under general anesthesia. After inserting a 6-F catheter sheath via femoral artery puncture, systemic heparinization was administered (50–70 IU/kg bolus, followed by 1,000 IU/h infusion). A 6-F guiding catheter was advanced to the petrosal ICA. Using roadmap guidance, a 0.021-inch stent microcatheter was navigated into the distal middle cerebral artery via a 0.014-inch micro guidewire. The Echelon-10 microcatheter was then positioned in the aneurysm sac. The EP-VRD2 was delivered and deployed using the “pushing over outer curve” technique, and a semi-jailing technique was applied (29).

Immediate follow-up angiography was performed to assess aneurysm embolization. Dyna CTA, with a 1:4 diluted contrast medium, evaluated stent marker positions and WA. If poor WA was detected, two methods were employed: advancing the stent microcatheter through the lumen to open the stent by tension or using a micro guidewire to massage and further open the stent. In the 12 cases where ISA was ultimately identified, these corrective maneuvers were attempted but failed to achieve complete apposition in 10 cases (83.3%).

### Clinical and imaging follow-up

Imaging follow-up was performed using DSA, with long-term follow-up extending beyond 12 months. Occlusion rates were assessed using the Raymond–Roy occlusion classification (RROC): I (complete occlusion), II (near-complete occlusion), and III (partial occlusion) (30). Classifications II and III were considered recurrences.

Procedure-related complications included IAs rupture and thromboembolic events. Clinical follow-up, conducted via outpatient visits or phone, used the modified Rankin Scale (mRS) to assess outcomes: scores of 0–2 indicated good outcomes, while 3–6 indicated poor outcomes.

### Stent conformability and wall apposition

**Figure 1:**
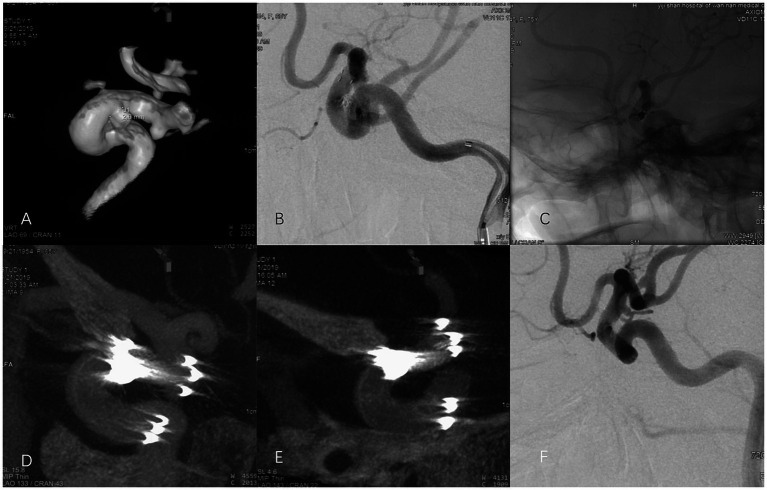
Representative images of ISA in a 62-year-old female with an unruptured right OSA treated with an EP-VRD2 stent. **(A)** 3D reconstruction of DSA showing the right OSA. **(B, C)** Immediate postoperative angiography revealed complete occlusion (RROC I), with **(B)** subtraction and **(C)** un-subtraction images. The stent size was 4.0 × 23 mm. **(D)** Dyna CTA showed a “CS” between the outer arc of the stent and the curved ICA siphon, with asymmetric proximal markers. **(D)** Dyna CTA showed a “CS” between outer arc of the EP-VRD2 and curved ICA siphon after initial deployment. The proximal markers were asymmetric and the distal markers were symmetric. **(E)** A small CS on the outer arc of the stent persisted on Dyna CTA even after attempts at stent massage with a micro-guidewire. **(F)** DSA after 17 months indicated complete occlusion.

Dyna CTA was performed immediately after stent deployment using a standardized protocol on the Artis Zee Floor system (Siemens, Germany). Acquisition parameters included: 360° rotation over 20 s, 109 kV tube voltage, 280 mA tube current, and a diluted contrast medium (Iodixanol 350 mgI/mL, 1:4 with saline) injected at 1.5 mL/s. Images were reconstructed with a slice thickness of 0.5 mm using a high-resolution kernel (Hr20) on the Syngo X Workplace (VB15, Siemens, Germany). ISA was defined by centering curl, luminal loss from ovalization, or lack of contact between the stent arcs and the vessel wall, with contrast medium flow outside the stent, known as the “crescent sign (CS)” by Heller et al. (10) (Figure 1).

The CA was defined as the stent’s inner and outer arcs maintaining contact with the vessel wall on Dyna CTA, without centralized curl or luminal loss from ovalization, regardless of marker symmetry at the stent ends.

ISA was defined broadly as any lack of complete contact between the stent and the vessel wall, which included but was not limited to: (1) the classic “crescent sign” (CS), characterized by contrast medium flow outside the stent arc (10) (Figure 1); and (2) other morphological signs such as centralized stent curling or luminal ovalization without evident contrast extravasation. This distinction is important because not all ISA manifests radiographically as a CS.

### Measurement of morphological characteristics

**Figure 2:**
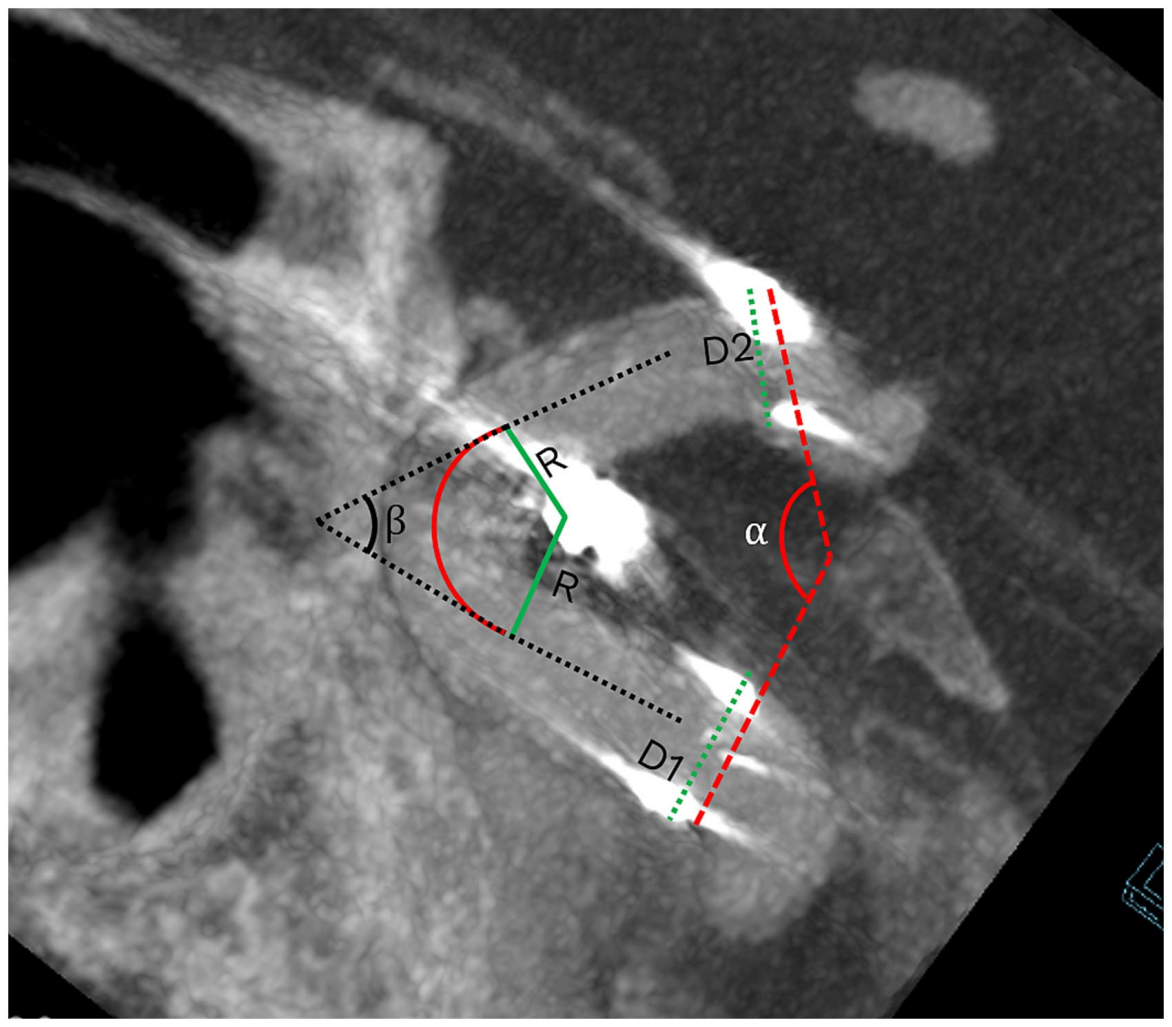
The definitions of morphological characteristics. Stent-subtended arc angle (SAA) = *α*; artery angle (AA) = *β*; parent vessel diameter (PVD) = (D1 + D2)/2; curvature radius (CR) = R.

Morphological measurements were made at the workstation post-stent and coil implantation, reconstructed using 3D-DSA with Syngo X Workplace. Data included stent-subtended arc angle (SAA), parent vessel diameter (PVD), curvature radius (CR), and artery angle (AA). AA was defined as the angle formed by the midline of the parent vessel on either side of the curve, measured on the plane of maximum curvature. CR was defined as the radius of the circle best fitting the inner curve of the vessel segment subtended by the stent (11). Detailed illustrations are provided in Figure 2. Two experienced neurosurgeons, blinded to clinical and apposition data, performed all measurements independently.

### Statistical analyses

Data were tested for normality using the Shapiro–Wilk test. Normally distributed data were expressed as mean ± standard deviation, and non-normally distributed data as median and interquartile range. Differences between CA and ISA groups were analyzed using the independent *t*-test or Mann–Whitney *U*-test. Categorical variables were compared using the continuity-corrected Chi-square test. Variables with a *p*-value < 0.05 in univariate analysis or deemed clinically relevant were included in a subsequent binary logistic regression model to identify independent predictors of incomplete stent apposition. ROC analysis identified cut-off values, with a *p*-value < 0.05 considered statistically significant. Statistical analysis was performed using SPSS 20.0 (SPSS Inc., Chicago, Illinois, USA) and MedCalc 15.0 (MedCalc Software bv, Ostend, Belgium).

## Results

### Clinical and aneurysm characteristics

A total of 104 patients with OSAs treated with EP-VRD2 were reviewed, including 30 with multiple aneurysms. Among the 104 OSAs, 32 (30.77%) involved the origin of the ophthalmic artery. The cohort consisted of 26 males and 78 females, aged 33–83 years (mean 59.17 ± 11.52). Sixteen patients had subarachnoid hemorrhage (Hunt-Hess grades I: 2, II: 12, III: 2). Comorbidities included hypertension (38 patients, 36.59%), diabetes mellitus (10 patients, 9.62%), and cerebral infarction (14 patients, 13.46%). Among the 88 patients (84.62%) with unruptured OSAs, 20 (19.23%) were asymptomatic, while others reported symptoms such as headache (19.23%), dizziness (30.77%), and blurred vision (2.88%). Ten patients (9.62%) had prior IA surgeries.

The size of OSAs ranged from 1.3 mm to 13.0 mm, with an average of 3.82 mm. Of these, 92 (88.46%) were less than 7 mm, 8 (7.69%) were 7–10 mm, and 4 (3.85%) were larger than 10 mm. The mean dome-to-neck ratio was 1.18 ± 0.30, with 4 aneurysms (3.85%) having a ratio ≥2 and 100 (96.15%) < 2. In terms of neck width, 36 aneurysms (34.62%) had a wide neck (≥4 mm), and 68 (65.38%) had a narrow neck (<4 mm) (Table 1).

We also compared demographics and aneurysm characteristics between the CA and ISA groups (Table 2). No statistically significant differences were observed in age, sex, comorbidities (hypertension, diabetes, cerebral infarction), proportion of multiple aneurysms, aneurysm size, neck width, proportion of wide-neck aneurysms, or dome-to-neck ratio (all *p* > 0.05), indicating that the two groups were well-balanced in terms of baseline features.

**Table 2:** Baseline characteristics of ISA vs. CA groups.

Parameter	CA group (n = 92)	ISA group (n = 12)	P
Age (mean ± SD)	58.9 ± 11.7	61.2 ± 10.8	0.51
Female (*n*, %)	70 (75.0%)	8 (66.7%)	0.71
Hypertension (*n*, %)	33 (35.9%)	5 (41.7%)	0.75
Diabetes mellitus (*n*, %)	9 (9.8%)	1 (8.3%)	>0.99
Cerebral infarction (*n*, %)	12 (13.0%)	2 (16.7%)	0.66
Multiple aneurysms, (*n*, %)	26 (28.3%)	4 (33.3%)	0.74
Aneurysm size (mm)	3.78 ± 2.91	4.12 ± 2.45	0.68
Neck width (mm)	3.58 ± 1.80	3.92 ± 1.88	0.52
Wide neck (≥4 mm), (*n*, %)	31 (33.7%)	5 (41.7%)	0.75
Dome-to-neck ratio	1.17 ± 0.29	1.21 ± 0.34	0.65

CA, complete apposition; ISA, incomplete stent apposition.

### Perioperative complications and clinical outcomes

EP-VRD2 stents of 23 mm and 30 mm were used to cross the ICA siphon bend, with a 78:26 ratio. All stents were successfully placed. Immediate postoperative angiography showed RROC I in 76 OSAs (73.08%), RROC II in 24 (23.08%), and RROC III in 4 (3.84%) (Table 3).

**Table 3:** Procedural details and outcomes.

Parameter	Results
Stent length (mm)	
23	78
30	26
Stent markers	
Prox.	S:A (64:40)
Distal	S:A (78:26)
ISA	12 (11.54%)
CS	9 (8.65%)
Other signs	3 (2.89%)
PVD (mm)	4.65 ± 0.45
AA (°)	58.43 ± 17.14
CR (mm)	4.20 ± 0.99
SAA (°)	105.72 ± 22.53
Initial angiogram results	
RROC I (*n* (%))	76 (73.08%)
RROC II (*n* (%))	24 (23.08%)
RROC III (*n* (%))	4 (3.84%)
Follow-up angiogram results	90 (86.5%)
RROC I (*n* (%))	86 (95.56%)
RROC II (*n* (%))	3 (3.33%)
RROC III (*n* (%))	1 (1.11%)
Complications	
Thromboembolic	3
Hemorrhagic	0
Clinical follow-up time	22.15 ± 9.26
MRS	
0–2	104
3–5	0

A, asymmetrical; S, symmetrical; ISA, incomplete stent apposition; CS, crescent sign; PVD, parent vessel diameter; AA, artery angel; CR, curvature radius; SAA, stent-subtended arc angle; RROC, Raymond–Roy occlusion classification; MRS, modified Rankin score.

There were 3 thromboembolic events (3.84%) during the perioperative period. Two occurred in the ISA group (2/12, 16.7%) and one in the CA group (1/92, 1.1%). One occurred during the procedure, where the patient developed stent thrombosis during the procedure, resolved by a 5-ml bolus of tirofiban (a platelet glycoprotein IIb/IIIa receptor inhibitor) into the stent catheter. One patient developed left hemiplegia 24 h post-procedure, treated with a 24-h tirofiban infusion, which improved symptoms. MRI later revealed an acute cerebral infarction in the right hemisphere. Another patient developed right hemiplegia, treated similarly. Both were discharged without significant symptoms, with an mRS score of 0–2. There was no significant difference in final mRS distribution between the two groups. There were no intraoperative bleeding complications. The procedural details and outcomes are displayed in Table 3.

### Follow-up results

Ninety patients (86.5%) were followed up with DSA over 12–40 months (average 19.91 ± 7.16 months). DSA images showed RROC I in 86 patients (95.56%), RROC II in 3 patients (3.33%), and RROC III in 1 patient (1.11%), with no statistically significant difference between the ISA and CA groups. Mild recurrence was observed in one patient, with no further operation needed. No stent thrombosis, malposition, new neurologic deficits, or deaths occurred during the follow-up period (average 22.15 ± 9.26 months). All 12 patients identified with immediate postoperative ISA completed follow-up DSA (mean 18.5 ± 6.2 months). All OSAs remained adequately occluded (RROC I or II).

### Stent conformability and wall apposition

**Figure 3:**
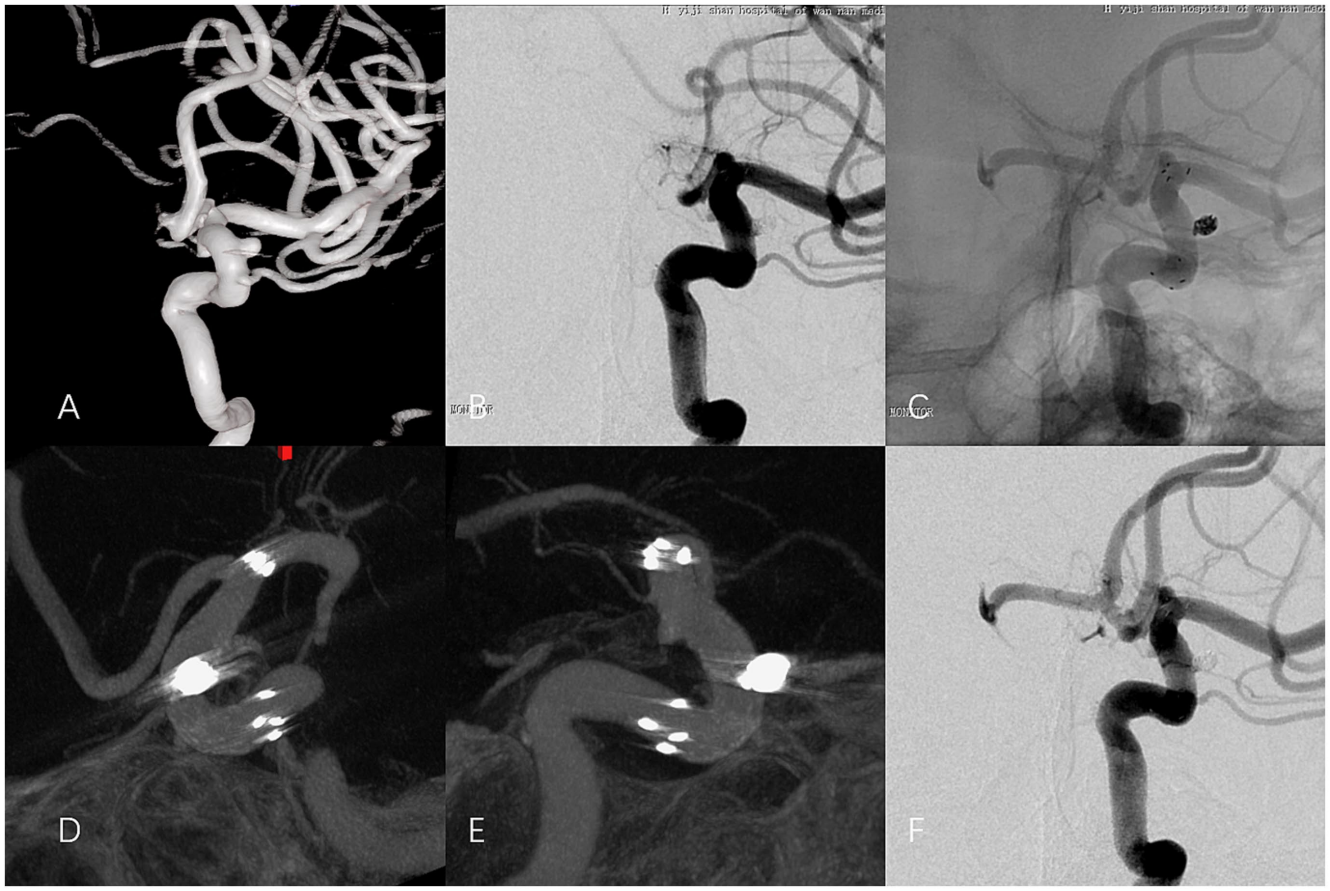
Representative CA images of an unruptured left OSA in a 71-year-old female treated with an EP-VRD2 stent. **(A)** 3D reconstruction of DSA showing the left OSA. **(B, C)** Immediate postoperative angiography revealed complete occlusion, with **(B)** subtraction and **(C)** un-subtraction images. The stent size was 4.0 × 30 mm. **(D, E)** Dyna CTA projections showing CA of the stent on both the inner and outer arcs in the curved ICA siphon. **(F)** DSA at 19 months confirmed complete occlusion.

All 104 patients underwent Dyna CTA to confirm stent WA. CA was observed in 92 patients (Figure 3). ISA was identified in 12 patients (11.54%). Among these, the classic CS was observed in 9 patients (8.65% of the total cohort), while the remaining 3 ISA cases (2.89%) exhibited other forms of incomplete apposition without a definitive CS.(Table 3).

**Figure 4:**
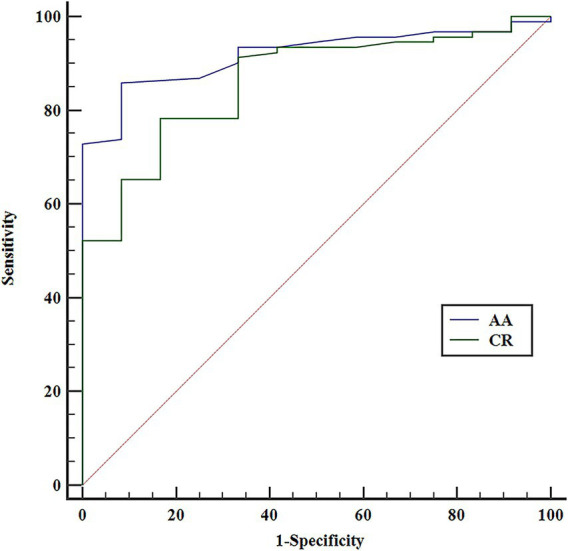
Receiver operating characteristic (ROC) curves for all the independent risk factors.

The mean PVD, AA, CR, and SAA for OSAs treated with EP-VRD2 were 4.65 ± 0.45 mm, 58.43 ± 17.14°, 4.20 ± 0.99 mm, and 105.72 ± 22.53°, respectively (Table 3). Significant differences in CR (*p* < 0.001) and AA (*p* < 0.001) were observed between the ISA and CA groups (Table 4). In the binary logistic regression analysis, both smaller CR (odds ratio [OR] = 0.22, 95% confidence interval [CI]: 0.08–0.61; *p* = 0.004) and smaller AA (OR = 0.88, 95% CI: 0.79–0.98; *p* = 0.022) were independently associated with ISA (Table 5). The cut-off values for AA and CR were 44° (AUC = 0.89) and 3.58 mm (AUC = 0.85) (Figure 4). The ISA group had smaller CR (3.16 ± 0.52 mm vs. 4.34 ± 0.95 mm) and AA (36.42 ± 8.23° vs. 61.30 ± 15.87°) compared to the CA group. No significant differences were found in stent length (*p* = 0.73), PVD (*p* = 0.21), or SAA (*p* = 0.06).

**Table 4:** Comparison of complete apposition and incomplete apposition.

Parameter	CA	ISA	P
Number of cases	92	12	
Stent length (23:30)	68:24	10:2	0.73
PVD (mm)	4.63 ± 0. 45	4.97 ± 0.56	0.21
SAA (°)	104.2 2 ± 22.48	117.25 ± 22.20	0.06
AA (°)	61.30 ± 15.87	36.42 ± 8.23	<0.001
CR (mm)	4.34 ± 0.95	3.16 ± 0.52	<0.001

CA, complete apposition; ISA, incomplete stent apposition; PVD, parent vessel diameter; SAA, stent-subtended arc angle; AA, artery angel; CR, curvature radius.

**Table 5:** The result of binary logistic regression analysis.

Parameter	*p* value	OR	95% CI
AA (°)	0.004	0.22	0.08–0.61
CR (mm)	0.022	0.88	0.79–0.98

*p* < 0.05 was considered statistically significant.

## Discussion

This study demonstrates that EP-VRD2 stents are safe and effective for treating OSAs, with good clinical outcomes and high occlusion rates. However, ISA was observed in cases involving sharply curved ICA siphons, particularly when the AA was less than 44° and the CR was less than 3.58 mm.

With advancements in interventional embolization materials and techniques, endovascular treatment has become the preferred approach for managing OSAs. Previous studies have reported immediate complete occlusion rates on DSA ranging from 9 to 72.6% following endovascular treatment of OSAs, with recurrence rates during follow-up ranging from 2 to 26% (12–14). Our results showed immediate occlusion and recurrence rates of 73.08 and 2.23%, respectively, which are consistent with the early outcomes reported in a recent preliminary study of EP-VRD2 for paraclinoid aneurysms (15). Our study, with a larger sample and longer follow-up, extends these findings. Furthermore, our results contribute to the growing real-world profile of EP-VRD2, complementing comparative evaluations performed in other clinical contexts (16). However, our immediate postoperative angiography results were slightly higher than previously reported. This discrepancy may be due to the EP-VRD2, a closed-loop stent that can be repositioned at any time and effectively ovalizes in curved vessels, helping to retain coils within the aneurysm and resulting in a high rate of immediate occlusion. Additionally, our study reported a relatively low recurrence rate, which could be attributed to two factors. First, the follow-up period of at least 12 months allowed sufficient time for endothelialization, likely contributing to the low recurrence rate. Second, the EP-VRD2’s design enhancements for curved vessels, particularly in the siphon segment, may have contributed to the lower recurrence rate.

Regarding clinical outcomes and perioperative complications, Ni et al. reported a 3.6% perioperative complication rate and a 99% good long-term outcome rate in their experience with 122 paraclinoid aneurysms treated with open-cell stents, with no procedure-related mortality observed (14). Wang et al. reported a perioperative complication rate of 4.3% during the endovascular treatment of 142 paraclinoid aneurysms, with complications occurring during the embolization process. The good outcome rate was 98% during the follow-up period (3). Our study reported a 3.84% perioperative complication rate, all due to thromboembolic events, with all patients achieving good treatment outcomes. These results are consistent with those of previous studies.

Although the EP-VRD2 stent was safe and effective for treating OSAs in the long term, ISA in the curved ICA siphon bends—particularly in cases with a small CR (<3.58 mm) and AA < 44°—cannot be overlooked. The adverse effects of ISA include intraoperative stent thrombosis and delayed postoperative cerebral infarction (7, 10, 11). The likely reason is that slow blood flow in the gap between the stent surface and the vessel wall can contribute to these complications. Second, ISA may be associated with or exacerbate stent malposition during or after the operation (17–19). Third, ISA might delay or hinder intimal formation (20). This phenomenon could affect the healing of IAs and lead to aneurysm recurrence (21). In our series, two of the three thromboembolic events occurred in the ISA subgroup (2/12, 16.7%), while only one occurred in the CA group (1/92, 1.1%). This pattern suggests that incomplete wall apposition may create areas of flow stagnation that theoretically increase thrombotic risk. Clinically, all three events were managed acutely with tirofiban, and all affected patients achieved a good recovery (mRS 0–2). Angiographically, occlusion rates at follow-up did not differ significantly between the groups. These observations, combined with the anatomical risk factors identified, lead to practical considerations for case management.

Our findings provide actionable anatomical thresholds (AA < 44°, CR < 3.58 mm) that should inform pre-procedural planning and intraoperative strategy. For treatment selection, for OSAs located in an ICA siphon segment with AA and CR below these cut-offs, the operator should be aware of a significantly higher risk of ISA with EP-VRD2. In such cases, alternative strategies should be strongly considered, including: (1) selecting a stent with potentially superior conformability in tighter curves (e.g., certain open-cell or braided stents, pending further comparative evidence); (2) opting for a FD if the anatomy is suitable and the risk of covering the ophthalmic artery is acceptable; or (3) considering microsurgical clipping for suitable surgical candidates. Regarding procedural strategy, if EP-VRD2 is deployed in a high-risk anatomy, we recommend: (1) Pre-procedural measurement of AA and CR on 3D-DSA to assess the risk. (2) Intraoperative vigilance: immediate post-deployment Dyna CTA is crucial to identify ISA. (3) Aggressive corrective maneuvers if ISA is detected: as described in our Methods, advancing the microcatheter to apply tension or using a micro-guidewire to “massage” the stent can improve apposition. (4) Enhanced antiplatelet management: for patients with residual ISA despite corrective efforts, consider more intensive perioperative antiplatelet monitoring or a short-term intravenous glycoprotein IIb/IIIa inhibitor infusion in the immediate post-procedure period.

However, our findings must be interpreted with caution. The number of ISA cases is small (n = 12), and thrombotic events were rare overall (n = 3). Therefore, we cannot draw a definitive statistical conclusion about a causal link between ISA and thromboembolism from this dataset alone. At the same time, our experience indicates that even if ISA occurs in difficult anatomies, such as tight ICA siphon bends, vigilant perioperative monitoring and immediate intervention can successfully manage acute thrombotic complications and lead to favorable outcomes.

Our findings on ISA and its predictors can be further contextualized by prior studies on stent conformability. In an *in vivo* study, Heller et al. found that the ISA rate of EP-VRD in the treatment of IAs was 19 out of 39 cases (49%) using 3 T-MRA and flat-panel CT. This was associated with large PVD, large SAA, and small CR (11). Kono et al. demonstrated *in vitro* experiments that the EP-VRD2, an upgraded version of the EP-VRD designed to WA in curved vessels, had better WA than the original EP-VRD (8). However, ISA persists in curved vessels with a small CR and AA, such as the ICA siphon. In a small-sample in vivo study, Herweh et al. reported two cases of ISA following EP-VRD2 deployment, both occurring in the ICA siphon with a small CR and AA (22). In our large-sample study, the EP-VRD2, designed to treat OSAs with a curved ICA siphon, showed an ISA rate of 12 out of 104 cases (11.54%), which is significantly lower than the 49% ISA rate reported for the EP-VRD in previous studies (11). We also found that ISA tends to be associated with smaller CR and AA in the EP-VRD2. In an in vitro study, Ebrahimi et al. observed that the EP-VRD began to flatten and kink when the AA was smaller than 90°; the smaller the curvature, the more severe the deformation (23). In our study on the EP-VRD2, we found that the cut-off value for AA was 44°, significantly lower than the 90° observed in previous studies. To some extent, AA reflects the radius of curvature. We were the first to identify a cut-off value for CR in the EP-VRD2 at 3.58 mm. However, Chen et al. found that ISA in the EP-VRD2 was associated with a stent length of 23 mm *in vivo* (9). Although their study did not show a significant relationship between the ISA and CA groups in terms of CR and AA, they reported an ISA rate of 16% (4/25), which is higher than the 11.54% we observed. Their results differed from ours for several possible reasons. Firstly, the total number of patients in their study was relatively small, with only 21 EP-VRD2 stents deployed through ICA siphon bends. Such a small sample size could lead to inaccurate results. Secondly, the AA in our study was smaller than that reported by Chen et al. A small sample size combined with a larger AA could easily lead to an underestimation of the ISA rate.

### Limitations

Our study has some limitations. First, this was a retrospective study without a prospective sample size calculation, which may limit the generalizability of the findings. The 104 OSAs treated with EP-VRD2 in this study represent the largest sample size to date; however, this was a single-center study. This retrospective, single-arm design is inherently susceptible to selection bias, as patient selection and treatment allocation followed a non-randomized institutional protocol. Furthermore, the absence of a control group limits comparative conclusions about the relative performance of EP-VRD2. Future multicenter studies with larger cohorts are needed to validate our findings. Second, Dyna CTA was not systematically repeated during angiographic follow-up, particularly for the 12 patients with initial ISA. Therefore, we could not evaluate the long-term morphological behavior of the stent within the parent vessel, which limits our understanding of the natural history of stent apposition over time. Additionally, the assessment of ISA on Dyna CTA, while based on established morphological signs (e.g., CS), involves a degree of subjective interpretation. We did not perform a formal inter-observer reliability analysis for ISA identification, which is a methodological constraint. The follow-up duration, while exceeding 12 months, may still be insufficient to capture very late ISA-related complications, such as delayed thrombosis or recurrence occurring years after implantation. Third, for unruptured OSAs, we also used new FDs for treatment. According to previous studies, the overall occlusion rate for IAs treated with FDs ranged between 85.0 and 96%, and the permanent morbidity rate ranged between 0 and 9.4% (24–28). Traditional endovascular treatment with the EP-VRD2 stent can achieve satisfactory results and may still be more cost-effective. However, most of the OSAs in our study were small to medium-sized aneurysms. For large and giant OSAs, the outcomes might differ. It is essential to stratify OSAs by size and study them separately in the future. This approach has the potential to yield more accurate and tailored results. Another consideration is the potential for attrition bias. Not all enrolled patients completed the scheduled imaging follow-up (90/104, 86.5%), and the clinical characteristics or outcomes of those lost to follow-up are unknown, which could influence the reported long-term efficacy and safety rates. Finally, the generalizability of our findings is constrained by the single-center design and the specific patient population treated at our institution. Aneurysm morphology, surgical expertise, and perioperative management protocols may vary across centers, potentially affecting outcomes. Fourth, inter-observer reliability for morphological measurements was not formally assessed with intraclass correlation coefficients, which may affect measurement consistency. Additionally, the proposed anatomical cut-off values for AA and CR are based on a limited number of ISA cases and should be considered exploratory until validated in larger, multicenter studies.

## Conclusion

To our knowledge, our study demonstrates that the EP-VRD2 is safe and effective for the long-term treatment of OSAs. However, ISA remains a concern in sharp ICA siphon bends, particularly when the AA is less than 44° or the CR is less than 3.58 mm. These findings may help in selecting the most appropriate stents for use in ICA siphon bends.

## Data Availability

The datasets presented in this study can be found in online repositories. The names of the repository/repositories and accession number(s) can be found in the article/supplementary material.
